# Improved housing versus usual practice for additional protection against clinical malaria in The Gambia (Roo*Pf*s): a household-randomised controlled trial

**DOI:** 10.1016/S2542-5196(21)00002-4

**Published:** 2021-04-08

**Authors:** Margaret Pinder, John Bradley, Musa Jawara, Muna Affara, Lesong Conteh, Simon Correa, David Jeffries, Caroline Jones, Balla Kandeh, Jakob Knudsen, Yekini Olatunji, Elisa Sicuri, Umberto D'Alessandro, Steve W Lindsay

**Affiliations:** a Department of Biosciences, Durham University, Durham, UK; b Medical Research Council Unit The Gambia at the London School of Hygiene & Tropical Medicine, Banjul, The Gambia; c Bernhard Nocht Institute for Tropical Medicine, Hamburg, Germany; d London School of Economics and Political Science, London, UK; e School of Public Health, Imperial College London, London, UK; f KEMRI-Wellcome Trust Programme, Kilifi, Kenya and Centre for Tropical Medicine and Global Health, Nuffield Department of Medicine, University of Oxford, Oxford, UK; g Department of Disease Control, London School of Hygiene & Tropical Medicine, London, UK; h MRC Tropical Epidemiology Group, London School of Hygiene & Tropical Medicine, London, UK; i National Malaria Control Programme, Banjul, The Gambia; j Royal Danish Academy - Architecture, Design, Conservation, Copenhagen, Denmark; k ISGlobal, Hospital Clínic, Universitat de Barcelona, Barcelona, Spain

## Abstract

**Background:**

In malaria-endemic areas, residents of modern houses have less malaria than those living in traditional houses. We aimed to assess whether children in The Gambia received an incremental benefit from improved housing, where current best practice of insecticide-treated nets, indoor residual spraying, seasonal malaria chemoprevention in children younger than 5 years, and prompt treatment against clinical malaria was in place.

**Methods:**

In this randomised controlled study, 800 households with traditional thatched-roofed houses were randomly selected from 91 villages in the Upper River Region of The Gambia. Within each village, equal numbers of houses were randomly allocated to the control and intervention groups using a sampling frame. Houses in the intervention group were modified with metal roofs and screened doors and windows, whereas houses in the control group received no modifications. In each group, clinical malaria in children aged 6 months to 13 years was monitored by active case detection over 2 years (2016–17). We did monthly collections from indoor light traps to estimate vector densities. Primary endpoints were the incidence of clinical malaria in study children with more than 50% of observations each year and household vector density. The trial is registered at ISRCTN02622179.

**Findings:**

In June, 2016, 785 houses had one child each recruited into the study (398 in unmodified houses and 402 in modified houses). 26 children in unmodified houses and 28 children in modified houses did not have at least 50% of visits in a year and so were excluded from analysis. 38 children in unmodified houses were recruited after study commencement, as were 21 children in modified houses, meaning 410 children in unmodified houses and 395 in modified houses were included in the parasitological analyses. At the end of the study, 659 (94%) of 702 children were reported to have slept under an insecticide-treated net; 662 (88%) of 755 children lived in houses that received indoor residual spraying; and 151 (90%) of 168 children younger than 5 years had seasonal malaria chemoprevention. Incidence of clinical malaria was 0·12 episodes per child-year in children in the unmodified houses and 0·20 episodes per child-year in the modified houses (unadjusted incidence rate ratio [RR] 1·68 [95% CI 1·11–2·55], p=0·014). Household vector density was 3·30 *Anopheles gambiae* per house per night in the unmodified houses compared with 3·60 in modified houses (unadjusted RR 1·28 [0·87–1·89], p=0·21).

**Interpretation:**

Improved housing did not provide protection against clinical malaria in this area of low seasonal transmission with high coverage of insecticide-treated nets, indoor residual spraying, and seasonal malaria chemoprevention.

**Funding:**

Global Health Trials funded by Medical Research Council, UK Department for International Development, and Wellcome Trust.

## Introduction

There have been considerable gains in malaria control in sub-Saharan Africa, with prevalence halving and incidence of clinical disease falling by 40% between 2000 and 2015.^1^ Nevertheless, malaria remains a substantial public health problem in the region, with 213 million clinical cases and 380 000 deaths in 2018, and in many places malaria control has stalled.^2^ The reduction in malaria has been achieved largely by the massive deployment of vector control interventions, mainly insecticide-treated nets and indoor residual spraying. The future success of these interventions, however, is threatened by low coverage, old and damaged nets,^3^, ^4^, ^5^ and the growing problem of insecticide-resistant mosquitoes.^6^ There is thus a pressing need to develop supplementary interventions for malaria control, especially if the goal is elimination and then eradication.^7^

A systematic review of the literature from 1900 to 2013 and a meta-analysis of 53 studies provided epidemiological evidence that good housing protects against malaria.^8^ Residents of good homes (with improved water and sanitation, sufficient living area, and constructed from durable material) had 42% lower odds of malaria infection compared to traditional homes and a 54–65% lower incidence of clinical malaria. Similarly, a meta-analysis of 29 malaria surveys carried out in 21 sub-Saharan Africa countries between 2008 and 2015 found that modern housing was associated with a 9–14% reduction in the odds of malaria infection compared with traditional housing, a level of protection comparable to insecticide-treated nets.^9^ A meta-analysis of the literature reported the results from only two trials concluding that screening might reduce malaria infection.^10^ Three household randomised-controlled trials of house screening have been published. In The Gambia, screening doors and windows and closing the eaves, the major entry point for Africa's principal malaria vector *Anopheles gambiae*,^11^ halved anaemia prevalence in children.^12^ In Ethiopia, incidence of clinical malaria was 61% lower in screened houses than in control houses.^13^ In Kenya, the odds of malaria infection were 54% less in children living in houses with screened eaves than those without screening.^14^

**Research in context**

**Evidence before this study**

We searched MEDLINE and the Cochrane Library using the term “malaria” and one or more of the terms “housing”, “screening”, “malaria control”, and “vector control” for randomised controlled trials and controlled before-and-after intervention studies of housing interventions published between Jan 1, 1900, and Nov 15, 2020. A systematic review of the literature between Jan 1, 1900, and Dec 13, 2013, analysed 53 observational studies in a meta-analysis, which found 47% lower odds of malaria infection and 45–65% lower odds of clinical malaria in residents of modern houses (finished wall and roof materials) compared with traditional houses. An analysis of 29 malaria surveys in sub-Saharan Africa from 2008 to 2015, consisting of 284 532 children, found a 9–14% reduction in the odds of malaria infection in modern housing (finished wall, roof, and floor materials) compared with traditional housing. The meta-analyses are very-low quality to low-quality evidence, because even when socioeconomic status is adjusted for, there remains the possibility of residual confounding. A systematic review of house screening published in 2020, based on only two trials, reported evidence of protection of low to moderate certainty. To date, there have been three household-randomised controlled studies: the Gambian study showed that screened ceilings reduced the odds of anaemia by 49% and full screening on the doors and windows, and closing eaves (the gap between the top of the wall and the roof) reduced the odds of anaemia by 47%(moderate-certainty evidence); the Ethiopian study reported screened doors and windows reduced clinical episodes of malaria by 62% (low-certainty evidence); and the Kenyan study found that screening the eaves reduced the odds of malaria infection by 54% (low-certainty evidence).

In our study, the addition of a metal saddle-shaped roof replicated the transition in roof typology, from a thatched to a metal roof, seen in The Gambia and across sub-Saharan Africa. We closed the eaves, and the overhanging roof, because this is normal practice in The Gambia. Open eaves are the major route by which malaria mosquitoes enter houses; thus closing the eaves could reduce mosquito house entry, providing the screened doors are closed. To cool the metal-roofed houses we added two large screened windows under the eaves. Cooling the house at night could reduce the time people stay outdoors in the evening, thereby reducing exposure to outdoor-biting malaria mosquitoes, and increase the usage of bednets, because being too hot at night is a major reason given by people for not using a net. In hotter parts of sub-Saharan Africa, increasing metal roofing on a large scale, could result in mass killing of indoor-resting malaria mosquitoes during the day because the maximum temperatures of ventilated metal-roofed houses are several degrees hotter than thatched houses.

**Added value of this study**

This clinical trial is the first to assess the effectiveness of a ventilated screened metal-roofed house against clinical malaria compared with traditional housing, in an area of low seasonal transmission and high coverage of standard malaria control interventions—ie, insecticide-treated bednets, indoor residual spraying, seasonal chemoprevention, and prompt and effective treatment with antimalarials. In relation to malaria control, there was no additional benefit of adding a metal roof, filling the eaves, and house screening in this study, and our analysis shows that malaria incidence was higher in the intervention group.

**Implications of all the available evidence**

Although there is a growing body of work that shows that good housing is associated with less malaria than traditional houses, these studies provide weak evidence because they are potentially subject to bias. Although socioeconomic status was adjusted for in the analyses, the potential for residual confounding remains. There have been few randomised controlled studies to measure the direct effect of mosquito screening on malaria transmission. The three previous studies, one of which was in The Gambia, showed clinical protection and reductions in the number of malaria mosquitoes entering screened houses compared with traditional unscreened houses. Our study was done among the poorest residents of the poorest region in The Gambia. We found higher malaria incidence in children living in modified houses than traditional houses, due to damage to the screening, and possibly due to residents staying outside the house for longer in the evening and not closing their doors until late. In these communities, alternative methods of protection are needed to prevent malaria mosquitoes entering the house and to keep the house cool. For poorly constructed houses, using eaves ribbons impregnated with spatial repellent or insecticide-treated screened windows and eaves baffles could increase protection. For better-quality houses, adding industry-quality screened doors and windows to existing houses or building new types of screened houses, with or without eaves tubes, should be explored.

The hypothesis supporting housing as being protective against malaria in sub-Saharan Africa is based on the observation that 79% or more of malaria transmission occurs indoors at night;^15^ therefore, reducing the porosity of houses to malaria mosquitoes should reduce malaria risk.^16^ In many parts of sub-Saharan Africa, traditional thatched-roofed houses with open eaves are being replaced by metal-roofed houses with closed eaves.^17^ We hypothesised that well-ventilated screened houses with metal roofs and closed eaves would lead to fewer malaria mosquitoes entering houses, thus less clinical malaria. Our study was designed to determine whether there is additional benefit for protecting children against clinical malaria of well-ventilated screened houses over current best practice of using insecticide-treated nets, indoor residual spraying for all, and seasonal chemoprevention for children younger than 5 years.

## Methods

### Study design

A generalised randomised complete block study, with control and intervention households randomly assigned within villages, was done in 2016–17 in the Upper River Region of The Gambia (appendix pp 5–6). This is an area of open Sudanian savanna with moderate levels of malaria transmission during the rainy season, from June to December. The principal malaria vector, *A gambiae* sensu stricto, had moderate resistance to deltamethrin, a pyrethroid insecticide, but resistance was low in *Anopheles coluzzii* and *Anopheles arabiensis*.^18^

The study was done in accordance with the International Conference on Harmonisation Tripartite Guideline for Good Clinical Practice and the Declaration of Helsinki (2000), whichever afforded the greater protection to the participants. The study was approved by The Gambia Government/Medical Research Council Joint Ethics Committee on Oct 29, 2014, (reference: SCC 1390v3) and the School of Biological and Biomedical Sciences Ethics Committee, Durham University, Durham, UK on Dec 1, 2014 (reference: SBBS/EC/1401/Roo*Pf*s Sept 12, 2014). The full study protocol has been reported previously.^19^

### Participants

Before any study activity, sensitisation meetings were held between community leaders and project staff. Consenting villages were surveyed to identify suitable houses from December, 2014, to March, 2015. Suitable houses were defined as houses with a single room, a thatched roof, open eaves, and mud walls in good condition with at least one child aged 6 months to 1xs sleeping there. Villages with at least four suitable houses in different compounds were selected; 37 villages on the north bank of the river Gambia and 54 on the south bank met the study criteria. A census of the children in 800 houses in the 91 study villages was done in June, 2016, to produce a list of resident children aged 6 months to 13 years.

Before the start of the study, separate written informed consent was obtained from each house owner to join the study, receive the house intervention, and to place light traps indoors, and from parents or guardians to enrol their child into the study.

### Randomisation and masking

**Figure 1:**
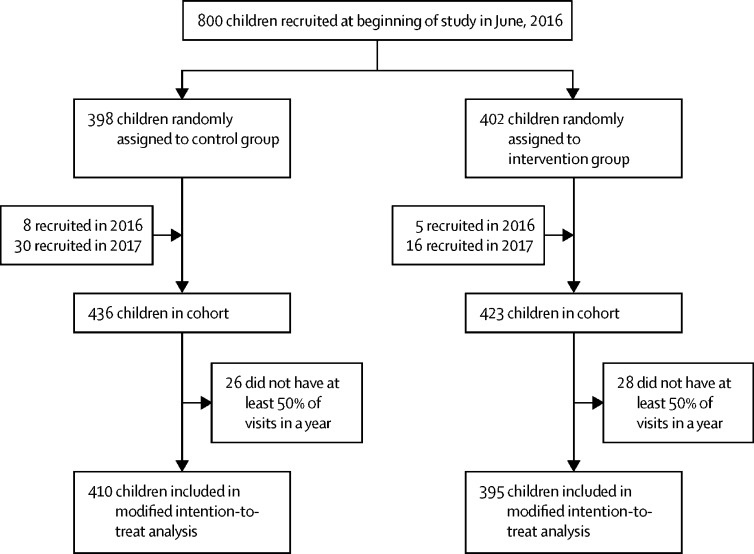
Trial profile

800 houses were randomly selected from 91 villages using census data. Within each village the houses were randomly allocated to the control and intervention groups. One child was randomly selected from each house and stratified by age (older or younger than 72 months) for each group. All randomisation was done using code written by DJ in MATLAB, from a database of census data. Within a village, half of the selected houses were allocated to each study group, with an additional house randomly allocated to a group if there was an odd number of houses. At least four houses (two per study group), were enrolled in each village. Stratified randomisation by village reduced the likelihood of chance imbalances between study groups. Additionally, data on potential confounders^20^ (child age, month, Fula ethnicity, and riverbank location) were collected and corrected for in the analysis of clinical malaria and mosquito entry. From the census list of the children in the study villages, one child per house was randomly selected for enrolment in the study cohort (figure 1) with roughly equal numbers of children aged 6 to 71 months and 72 to 156 months. When possible, children who left the study were replaced by another child, randomly selected from within the same house. Of the 800 houses, 120 were randomly selected for monthly light trap collections, balanced between groups and stratified by village and riverbank location (north or south). The randomisation was done electronically by DJ directly from the study database.

It was impossible to conduct this study in a fully masked manner. Observer bias was reduced where feasible. Blood films were read by microscopists masked to the intervention status of the participants. Bias arising from using mosquito collectors was reduced by using standard traps that do not rely on a fieldworker to collect specimens. Mosquitoes were examined and analysed by technicians who did not know the trap location. Datasets were unmasked once data critical for the listed endpoints had been locked.

### Procedures

**Figure 2:**
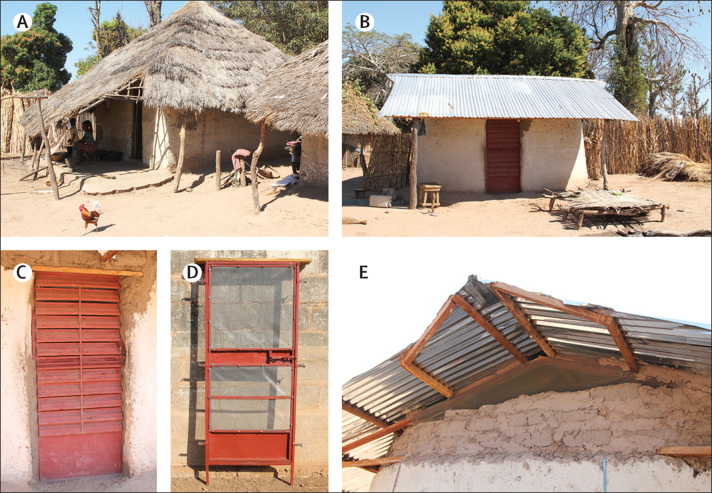
Study houses Unmodified, thatched-roofed house (A), modified, ventilated screened house with metal roof (B), fixed-louvered front door with mosquito screening behind the door (C), screened back door (D), and ventilated screened window in each gable end (E).

At baseline, enrolled houses had a single room, thatched roofs, open eaves, and mud walls in good condition and occupants provided with sufficient insecticide-treated nets (Olyset, Sumitomo Chemical, Japan) to cover all sleeping places in all enrolled houses in July, 2016, and by the National Malaria Control Programme (NMCP) in August, 2017, as part of the national mass campaign. In the intervention group, houses with thatched roofs were replaced with metal roofs, eaves were closed, metal fixed-louvered screened doors were installed at the front of the house, and a metal-screened door at the back and screening on the window was installed, if present (figure 2).^21^ The house modifications were made before June, 2016, and the condition of the intervention houses was assessed during and immediately after the modifications were complete and again in August, 2016, and July, 2017, with repairs done after the cross-sectional surveys. House owners in the intervention group were encouraged to report any damage or malfunctioning of the interventions to the nurse field assistants who visited twice per week. Houses in the control group, representing traditional houses, were not modified, so were left with thatched roofs and open eaves until the end of the study (the control group received house modifications after the end of the study in December, 2017). The condition of all houses was assessed at the end of the study in January, 2018. Interventions were installed from February, 2015, to June, 2016, and homeowners in both groups encouraged to keep their doors closed at night and sleep under an insecticide-treated net. Indoor residual spraying and seasonal chemoprophylaxis were also provided by the NMCP (the dates of which were recorded during the transmission seasons in 2016 and 2017).

Incidence of clinical disease was assessed by active case detection. The children in the study were visited at home twice per week and axillary temperature taken by trained nurse field assistants, for two malaria transmission seasons (June to December) in 2016 and 2017. A rapid diagnostic test (RDT; Paracheck Pf, Orchid Biomedical Systems, Goa, India) was done immediately if the axillary temperature was 37·5°C or more, if the child had had fever since the previous visit, or was feeling unwell. A blood sample was collected for thick films for *Plasmodium falciparum* detection only when the axillary temperature was 37·5°C or more. Presence of cough, a raised age-specific respiratory rate (>50 breaths per min for children younger 1 year, >40 breaths per min for children aged 1–5 years, and more than the mean breaths per min plus two standard deviations for observations in children older than 5 years) or chest indrawing was also assessed, because there was concern that closing the eaves would reduce ventilation in modified houses and increase respiratory infections. During the cross-sectional surveys (done in December, 2016, June, 2017, and December, 2017), a clinical examination was done on all study children, axillary temperature collected, and spleen size assessed. Children were fingerpricked for measurement of anaemia with a spectrophotometer (HemoCue, Ängelholm, Sweden) and an RDT was done. Thick blood films were examined by trained, experienced microscopists. Parasite counts were recorded per high-power field and 100 fields were counted before a slide was declared negative. Two slides were prepared from each individual and assessed separately by two experienced microscopists, with discrepancies resolved by a third.

To estimate potential exposure to malaria mosquitoes, mosquito collections were made using light traps from the Centers for Disease Control and Prevention, placed 1 m from the ground at the foot end of a study child's insecticide-treated net. The density of malaria vectors was assessed once a month from June to December in 2016 and 2017. Mosquitoes were identified by microscopy and counted. The presence of sporozoites in *A gambiae* sensu lato were identified by ELISA^22^ and species typed by PCR.^23^, ^24^

Indoor temperature was measured in 13 control houses and 15 intervention houses during the rainy season (Sept 7 to Dec 11, 2015). Recordings were made in houses in the same villages every 30 min with data loggers (Tiny tag, TG U 4500) positioned 1 m from the floor in the centre of the room, in one unmodified house and one modified intervention house in the same village on the same dates. Data handling complied with the relevant Medical Research Council Unit The Gambia Standard Operating Procedures. Electronic Case Report Form data were collected using OpenClinica and stored on a secure server. Paper Case Report Forms were double entered into a Microsoft Access database, which after entry were password protected and accessible by DJ, JB, and MP. After database lock, the trial data manager extracted an anonymised final analysis dataset into a passworded Microsoft Access database.

### Outcomes

The primary parasitological endpoint was the incidence rate of clinical malaria, with a clinical episode of malaria defined as axillary temperature of 37·5°C or more and a parasitaemia of any density. The primary entomological endpoint was the number of *A gambiae* sensu lato collected per light trap per night. Secondary endpoints included a broader definition of clinical malaria—ie, a positive blood slide or a positive RDT and either documented fever (axillary temperature ≥37·5°C), reported fever since previous visit, or feeling unwell; prevalence of malaria parasites; prevalence of mild anaemia (>80–110 g/L), moderate anaemia (>50–80 g/L), severe anaemia (≤50 g/L), haemoglobin concentration, and prevalence of enlarged spleens. At each home visit for active case detection, children were asked if they had slept in the house the previous night.

Serious adverse events (SAEs) in children in the study were documented. The trial followed standard definitions for SAEs agreed by consensus of the Collaborating Centres of the WHO's International Drug Monitoring Centre (Uppsala, Sweden).

### Statistical analysis

We used computer-generated simulations to estimate power and found that with a 1:1 allocation ratio, one child per house followed up for 2 years would require 400 houses in each study group to detect a 35% reduction (the minimum we considered to be of public health importance) in the rate of malaria cases with over 80% power at the 5% significance level. For the entomological endpoint, the study was 80% powered to detect a 50% reduction at the 5% level of significance. Methods for sample size calculation have been described previously.^18^

Analysis was done with Stata version 15.0. Data analysis followed an analysis plan written before study completion. All analyses were done on a modified intention-to-treat basis in which all children with fewer than 50% visits in a year were not included. Data were censored for 4 weeks after a child had a documented malaria infection, and if the child had moved from the study house. If a child was absent more than half the scheduled visits in transmission season, their data were censored for that year. We compared between study groups the incidence rate of clinical malaria over the course of the transmission seasons (June to December) in 2016 and 2017, both separately and combined. Poisson regression with robust standard errors to account for repeat episodes for a child was used to compare the rates between groups, together with Kaplan-Meier plots showing time to first event by study group. An analysis adjusted for age, riverbank, month of year, and ethnicity was also carried out. The rate ratio comparing the mean number of female *A gambiae* sensu lato per light trap per night groups was calculated using negative binomial regression with a random effect for house, adjusting for ethnicity, riverbank, and burning incense (churai).

Parasite rates and density, anaemia prevalence and haemoglobin concentrations from the cross-sectional data were analysed by either logistic or linear regression according to whether the variable was binary or continuous. The rate of respiratory infection over the course of the two annual transmission seasons was analysed in the same way as clinical malaria.

Entomological inoculation rate (EIR) was estimated in each study group and is defined as the number of infective bites received per person during the transmission season, and was estimated as follows: EIR=HDM × SPR × *n* (where HDM is the household density of mosquitoes, which is estimated as the mean number of *A gambiae* sensu lato per light trap per night; SPR is the sporozoite rate; and *n* is the number of nights during the transmission season, July to December [*n*=6 × 30=180]).

Houses classified in good condition were those with all window and door screening intact and with the doors having no gaps around the edges when shut. We hypothesised that houses where the doors were kept closed during the day were more likely to have their doors shut at night, compared with houses where the doors were left ajar. Similarly, we considered that houses with more people would have their doors opened more frequently during the night than those with fewer people.

A Data Safety Monitoring Board reviewed the study procedures and results. The trial is registered at ISRCTN02622179.

### Role of the funding source

The funder of the study had no role in study design, data collection, data analysis, data interpretation, or writing of the report.

## Results

In 2014, 91 villages agreed to participate in the study and were enrolled. The NMCP provided insecticide-treated nets to all study villages in June, 2015, and August, 2017. In July, 2017, we distributed 1513 insecticide-treated nets to all study households. Insecticide-treated net coverage increased from 397 (52%) of 762 children in June, 2016, at the start of the rainy season, to 715 (94%) of 762 children by the end of July, 2016, and coverage remained high in 2017 with 659 (94%) of 702 children in December, 2017 (appendix p 1). Indoor residual spraying with bendiocarb (FICAM WP 80, Bayer, Germany) was implemented in November, 2016, (late in the malaria transmission season), with coverage of 245 (55%) of 445 children. In July, 2017, coverage was 662 (88%) of 755 children with pirimiphos methyl (Actelllic, Syngenta, UK). Children younger than 5 years received seasonal malaria chemoprevention with amodiaquine in September, October, and November in both years from the NMCP; in 2016, 192 (66%) of 292 children received one or more doses and in 2017, 151 (90%) of 168 children received one or more doses.

Among the 859 children recruited into the study, including replacements for the original cohort, 54 were absent for at least 50% of home visits in both years and were excluded from the analysis. Therefore, the final data set included 805 children, 410 in unmodified houses and 395 in modified houses (figure 1). The baseline characteristics of study children were similar in both groups, although there was a lower prevalence of malaria infection in the children living in modified houses than unmodified houses (table 1).

**Table 1:** Baseline characteristics of surveyed children*

		**Control group**	**Intervention group**
Sex			
	Female	47% (179/382)	49% (188/380)
	Male	53% (203/382)	51% (192/380)
Age, years		6 (4–8)	5 (3–8)
Ethnicity			
	Fula	64% (246/382)	64% (245/380)
	Mandinka	33% (127/382)	32% (121/380)
	Other	2% (9/382)	4% (14/380)
Living on north bank of river		57% (216/382)	56% (213/380)
Number of children aged 6 months to 13 years old		4 (3–6)	4 (3–5)
Slept under ITN previous night		54% (205/382)	51% (192/380)
*Plasmodium falciparum* parasite prevalence		5% (19/382)	2% (9/380)
Prevalence of mild anaemia, >80–110g/L		46% (172/377)	50% (190/376)
Prevalence of moderate anaemia, >50–80 g/L		7% (27/377)	7% (25/376)
Prevalence of severe anaemia, ≤50 g/L		<1% (1/377)	1% (3/376)
Haemoglobin, g/L		11·0 (10·0–11·8)	10·8 (9·8–11·7)
Prevalence of enlarged spleen		<1% (1/378)	0% (0/380)

Data are % (n/N) or median (IQR). ITN=insecticide-treated net.

* Data was not available for 16 children in the control group and 22 children in the intervention group.

**Figure 3:**
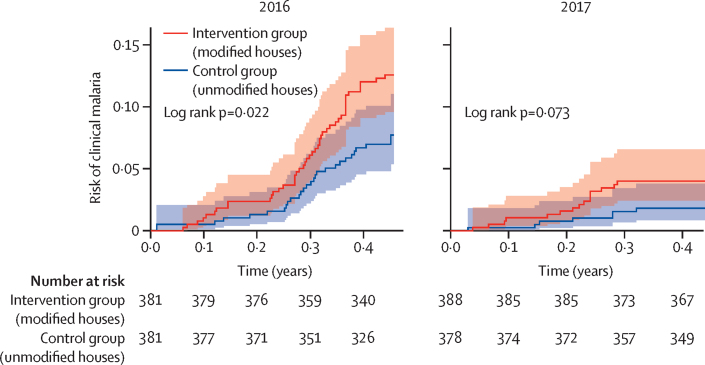
Time to first clinical malaria episode by study group

The 805 children included in the primary analysis were followed up for 654·6 child-years (330·1 in the control group and 324·5 in the intervention group) over two transmission seasons. There were 104 episodes of clinical malaria, 39 in children in the unmodified houses and 65 in the modified houses. The incidence rate of clinical malaria was 0·20 episodes per child-year in children in the modified houses and 0·12 per child-year in the unmodified houses, giving an unadjusted rate ratio (RR) of 1·68 (95% CI 1·11–2·55; p=0·014); the result remained significant after adjusting for age, riverbank, month of year, and ethnicity (appendix p 1). Incidence of clinical malaria was higher in 2016 than in 2017 but, in both years, it was higher in the modified-house group. When using the broader definition of clinical malaria, incidence rate was higher in the modified-house group, although less pronounced, and the difference between groups was not significant (table 2). Kaplan-Meier survival curves indicate that the rate of clinical cases increased towards the end of the malaria transmission seasons in both groups in 2016 but only in the intervention group in 2017 (figure 3). There were no significant differences between the groups in malaria prevalence, anaemia prevalence, haemoglobin concentration, or prevalence of enlarged spleens (table 3). Incidence of respiratory infections was also similar between the two study groups (unadjusted RR 1·03 [95% CI 0·74–1·42], p=0·87; table 2). Reported SAEs were similar in both study groups, with 20 children in the unmodified houses being hospitalised (three with severe malaria, nine with pneumonia, and eight with reasons not linked to the intervention) and 21 in the modified houses (five with severe malaria, seven with pneumonia, and nine with reasons not linked to the intervention).

**Table 2:** Comparison between groups on incidence of clinical malaria, malaria morbidity, and respiratory infections in the modified intention-to-treat population

	Rate of disease*		**Unadjusted RR (95% CI)**	**Adjusted RR (95% CI)**†	**p value**
	Unmodified houses	Modified houses			
**Clinical malaria incidence**‡					
Overall	0·12 (39/330)	0·20 (65/324)	1·68 (1·11–2·55)	1·75 (1·15–2·65)	0·0085
2016	0·19 (32/166)	0·30 (50/165)	1·57 (0·98–2·52)	1·65 (1·03–2·66)	0·039
2017	0·04 (7/164)	0·09 (15/159)	2·21 (0·91–5·38)	2·20 (0·90–5·37)	0·084
**Malaria morbidity incidence**§					
Overall	0·27 (88/330)	0·32 (105/324)	1·21 (0·88–1·65)	1·26 (0·92–1·71)	0·14
2016	0·40 (67/166)	0·44 (73/165)	1·09 (0·76–1·57)	1·16 (0·81–1·66)	0·42
2017	0·13 (21/164)	0·20 (32/159)	1·57 (0·89–2·75)	1·59 (0·90–2·79)	0·11
**Respiratory infection incidence**					
Overall	0·52 (169/325)	0·54 (173/321)	1·03 (0·74–1·42)	0·85 (0·65–1·11)	0·24
2016	0·78 (127/162)	0·81 (131/162)	1·03 (0·73–1·45)	0·84 (0·63–1·14)	0·26
2017	0·26 (42/163)	0·26 (42/159)	1·03 (0·60–1·77)	0·88 (0·54–1·44)	0·61

Data are rates (event/child-years) or mean (95% CI). RR=rate ratio.

* Number of events per child year.

† Adjusted for age, riverbank, month of year, and ethnicity.

‡ Clinical malaria incidence is based on axillary temperature of 37·5°C or more and a parasitaemia of any density.

§ Malaria morbidity incidence is a positive blood slide or rapid diagnostic test with documented or reported fever or feeling unwell.

**Table 3:** Comparison between study groups on measures of malaria transmission from cross-sectional surveys in the modified intention-to-treat population

	Survey 1*					**Survey 2**†					**Survey 3**‡				
	Unmodified houses (n=370)	Modified houses (n=370)	OR (95% CI)	RR (95% CI)	p value	Unmodified houses (n=361)	Modified houses (n=365)	OR (95% CI)	RR (95% CI)	p value	Unmodified houses (n=354)	Modified houses (n=348)	OR (95% CI)	RR (95% CI)	p value
*Plasmodium falciparum* prevalence	2% (8/370)	2% (7/370)	0·87 (0·31 to 2·43)	..	p=0·81	1% (5/361)	1% (2/365)	0·39 (0·08 to 2·04)	..	p=0·27	1% (3/354)	1% (2/348)	0·68 (0·11 to 4·07)	..	0·67
Prevalence of mild anaemia, >80 to 110 g/L	31% (113/368)	37% (136/370)	1·31 (0·97 to 1·78)	..	p=0·083	37% (135/361)	37% (132/364)	0·95 (0·70 to 1·29)	..	p=0·75	31% (108/354)	27% (97/348)	0·88 (0·64 to 1·22)	..	0·44
Prevalence of moderate anaemia (>50 to 80 g/L)	5% (20/368)	5% (17/370)	0·84 (0·43 to 1·63)	..	p=0·60	2% (7/361)	4% (13/364)	1·87 (0·74 to 4·75)	..	p=0·19	1% (5/354)	2% (7/348)	1·43 (0·45 to 4·56)	..	0·54
Prevalence of severe anaemia ≤50 g/L	<1% (1/368)	0% (0/370)	..	..	..	<1% (1/361)	<1% (1/364)	0·99 (0·06 to 15·92)	..	p=0·99	0% (0/354)	0% (0/348)	..	..	..
Mean haemoglobin, g/L	11·39	11·30	..	−0·10 (−0·36 to 0·17)	p=0·47	11·33	11·24	..	−0·08 (−0·30 to 0·14)	p=0·47	11·85	11·85	..	0·01 (−0·23 to 0·25)	0·96
Prevalence of enlarged spleen	<1% (1/368)	<1% (1/369)	1·00 (0·06 to 16·00)	..	p=1·00	<1% (1/361)	0% (0/364)	..	..	..	<1% (1/351)	0% (0/348)	..	..	..

Data are % (n/N), OR (95% CI), or RR (95% CI). OR=odds ratio. RR=rate ratio.

* Survey 1 was done in December, 2016.

† Survey 2 was done in June, 2017.

‡ Survey 3 was done in December, 2017.

A total of 4965 *A gambiae* sensu lato were caught in study houses on 1440 trapping nights, with most being *A arabiensis* (appendix p 3). There was a similar species composition in both study groups each year. Mosquito densities were slightly higher in modified houses (3·60 [SD 12·1] mosquitoes per house per night) compared to unmodified houses (3·30 [11·4] mosquitoes per house per night), but this was not significant (unadjusted RR 1·28 [95% CI 0·87–1·89], p=0·21). After adjusting for year, ethnicity, riverbank, and incense burning, there was no difference between study groups (1·23 [0·83–1·81], p=0·30; appendix p 2). There were no differences in sporozoite rates or EIRs between study groups (appendix p 3).

During the day, the maximum indoor temperature was 1·5°C hotter in modified than in unmodified houses (95% CI 0·7–2·2, p=0·0004). Between 2000 h and 2400 h, it was 0·7°C hotter in modified houses than unmodified houses (0·1–1·2, p=0·018). By midnight both house typologies had similar temperatures (appendix p 7).

In modified houses, there was extensive damage to the screening on the front doors (273 [75%] of 363), but less so on back doors (85 [24%] of 360), windows on the sides of the houses (20 [17%] of 115), and screening in the gable ends (14 [4%] of 357; appendix p 4). There were also gaps between the frame and front door (87 [24%] of 362), and the frame and back door (67 [19%] of 363) of modified houses. At the end of the trial, only 139 (35%) of 392 modified houses had intact screening and 81 (21%) of 392 modified houses had front and back doors that were able to close firmly. Clinical malaria incidence rates (adjusted RR 1·53 [95% CI 0·92–2·54], p=0·10) and the number of mosquitoes per night (1·47 [0·87–2·48], p=0·15) tended to be higher in intact modified houses than non-intact modified ones, although the differences were not significant. Incidence rates of clinical malaria (0·66 [0·31–1·39], p=0·28) and the number of mosquitoes per night (0·44 [0·27–0·74], p=0·0018) were lower in children in modified houses with closed doors than in modified houses with open doors. The number of occupants was not associated with the rate of clinical malaria episodes (p=0·41) nor density of mosquitoes (p=0·57). An unplanned analysis looking at differences between boys and girls among older children living in modified houses was done. Six (19%) of 32 boys aged 10 years or older lived in intact houses, compared with seven (47%) of 15 girls of the same age (p=0·046). In this group, boys were at more risk of clinical malaria (1·32 [0·39–4·48], p=0·65), but this was not significant.

## Discussion

In this rural region of The Gambia with moderate seasonal malaria transmission and high coverage of standard control interventions, living in a ventilated, screened house did not provide children with additional protection against clinical malaria. On the contrary, children sleeping in modified houses were at higher risk of clinical malaria than those sleeping in unmodified houses. This effect was consistent between study years and became more pronounced towards the end of each malaria transmission season. Nevertheless, the higher risk of clinical malaria was not associated with higher vector density in modified houses compared with unmodified ones. Additionally, no difference in secondary outcomes such as prevalence of malaria infection, anaemia, prevalence of enlarged spleens, and mean haemoglobin concentration was observed, which might simply reflect the prompt and effective treatment provided to all children with clinical malaria.

Our findings are surprising for two reasons. Firstly, in a previous trial in The Gambia, house screening was protective against malaria anaemia.^12^ Secondly, four multi-country reviews indicate that improved housing, compared with traditional housing, is protective against malaria.^8^, ^10^, ^25^, ^26^ One explanation is that our study site is not representative of other sites in sub-Saharan Africa. In an analysis of data from 29 surveys done between 2008 and 2015 in 21 sub-Saharan African countries,^25^ seven sites showed an increase, rather than a decrease, in the odds of malaria infection in children younger than 5 years living in modern housing compared with traditional houses. The Gambian site had the largest increased odds of all the surveys, for malaria infection both by microscopy (odds ratio [OR] 2·11 [95% CI 0·42–10·73]) and RDT (1·48 [0·61–3·59]). By contrast, for insecticide-treated nets, the Gambian site showed the greatest protection against malaria infections detected by microscopy (OR 0·29 [0·06–1·51]) and RDT (0·51 [0·25–1·04]). Although these results were not significant, they suggest that in rural Gambia, people prioritise bednet use to house screening as the primary method for reducing mosquito biting. This suggestion is supported by the high bednet coverage found in the Roo*Pf*s study. Unlike most countries in sub-Saharan Africa, The Gambia has a long history of bednet use, dating back at least to the early 1890s;^27^ thus our findings might be specific to this country and area.

The question why children in ventilated, screened houses had a higher incidence of clinical malaria than those in traditional houses remains. In 2016, the higher indoor temperatures experienced in the evening in metal-roofed screened houses compared with traditional houses,^28^, ^29^ might partly explain our findings. A hot house might have delayed the time children went to bed, exposing them to longer periods of time outdoors where they could be bitten by a mosquito, and might have reduced bednet use at night; particularly because this study included children older than 5 years, and children older than 8 years have greater autonomy in terms of deciding when they go inside to sleep. Although, reported bednet usage was similar between study groups, accurately measuring use in the field is challenging.^30^ In 2016, however, both roof types had similar temperatures before midnight.^29^ Hot houses at night are unlikely to improve malaria protection for individual households; however, they might be protective if used by entire communities. Although this might appear paradoxical, we have shown that the hotter metal-roofed houses, including the modified houses used in this study, reduced the survival of *A gambiae* resting indoors compared with those in traditional thatched houses.^29^ Reducing vector survival will disproportionately reduce the EIR.^31^, ^32^

We found similar numbers of *A gambiae* in modified and unmodified houses, indicating that the doors were not kept shut at night or that the door and window screening were so badly damaged they were no longer a barrier to mosquitoes, or both. In an ancillary study in The Gambia, we found that before midnight there was considerable amount of door opening in houses as people prepared for prayers and their evening meal.^33^ In some houses, people preferred to keep the doors open until late at night, showing that they had nothing to hide or, in a few cases, to allow good luck to enter. The screening on our doors and windows was easily damaged and gaps were common between the door and frame; this was probably the major route by which malaria mosquitoes entered intervention houses. The damage was more common in the homes of older boys, where there is little supervision from adults, compared with older girls, who usually sleep with an adult woman. Low levels of adherence have also been recorded in 5–19-year-olds when using insecticide-treated nets,^34^ highlighting the challenges faced protecting those with greatest reservoir of infection. In a pilot study, however, well-built, self-closing doors reduced mosquito house entry by 59–77% compared with the control houses.^33^ Clearly, better manufactured self-closing doors and windows with more durable screening are needed to improve mosquito control.

It would be erroneous to think that the traditional thatched-roof house represents the best typology for reducing malaria transmission. Traditional thatched-roofed houses have open eaves, the main entry point for *A gambiae*.^11^, ^28^ Moreover, today most eaves are closed in The Gambia, partly because of a public health campaign by the NMCP, which preceded the start of the Roo*Pf*s study. Our study cohort, therefore, consisted of individuals who ignored or were unaware of the campaign, which might have meant that the study group was less receptive to other behavioural changes, such as closing the doors at night, than other members of the community. Another contributory factor to people not closing their doors at night might have been the relatively low numbers of mosquitoes entering houses. An earlier study showed that people protected themselves against mosquitoes with physical barriers (untreated bednets) only when there were more than 20 mosquitoes entering a house at night.^27^ Nonetheless, closing the eaves of thatch houses will make the room 0·5°C hotter for the period before midnight.^28^ Across sub-Saharan Africa, thatched-roofed houses are declining, with metal-roofed houses becoming the new norm.^17^

Concern was raised that screening a house would reduce airflow and might exacerbate respiratory diseases in children. This is not the case in our study, because the incidence of respiratory illness was similar in both study groups. We also have evidence that ventilation in both groups of houses is similar.^35^

In the study villages, the levels of insecticide-treated net ownership and indoor residual spraying coverage were higher than those reached by many African countries, including The Gambia.^2^ In 2017, the incidence of malaria in study children was 90% lower than in 2016, probably due to the high level of malaria control activities and the shorter rainy season in 2017. Although there was high coverage of insecticide-treated nets in both years, in 2017 pirimiphos-methyl, a highly effective insecticide, replaced bendiocarb used in 2016, and was applied at the start of the rainy season in 2017, rather than late in the malaria transmission season in 2016. Moreover, seasonal malaria chemoprevention was used widely to treat children aged 6 months to 5 years in 2017.

This study has five main limitations. Firstly, the study was carried out in a subset of the population, the poorest households who were unable to afford metal-roofed houses, which might make generalisation difficult. Secondly, children in Gambian villages might occasionally sleep in other houses in the same village, and the house owner or dweller might not divulge this to not forfeit the benefits of the study. Thirdly, this study was unmasked and might be subject to differential behaviours of the field staff and participants. Fourthly, a study (Mmbando AS, Ifakara Health Institute, Tanzania, personal communication) suggests that the catching efficiency of light traps is greater in screened houses than control houses, because in houses with screened doors the light from the trap is seen by mosquitoes outside the house, unlike the solid doors of the control houses. If true, this scenario is likely to inflate mosquito collections in intervention houses relative to control ones. Fifthly, a cluster randomised study could have shown whether mass killing of malaria mosquitoes in intervention houses reduced malaria transmission compared with clusters of traditional houses.

A perfectly screened house, where the doors are infrequently opened, will reduce the entrance of malaria vectors. Our study, however, showed that in this region of The Gambia, modifying a traditional house to provide a metal roof and screening to the poorest of the poor did not provide protection against clinical malaria compared with those living in traditional thatched-roofed houses. In addition to standard best practice, novel solutions are needed to improve protection in the home. For the poorest households, using indoor residual spraying, eaves ribbons impregnated with spatial repellent,^36^ or insecticide-treated window screens and eave baffles^37^ could increase protection. For less poor households, living in better built houses,^38^ using industry-quality screened doors and windows properly fitted^33^ to modify existing houses or building from scratch new types of screened houses, with or without eaves tubes,^39^ should also be explored. Housing interventions to keep out mosquitoes and keep the house cool need to be tailored to local climates and conditions, and built with durable materials to high standards. Importantly, to achieve the UNs' Sustainable Development Goal 11, to make settlements inclusive, safe, resilient and sustainable, rural communities in sub-Saharan Africa require access to building innovations, and microloans for building improvements and an understanding of the behavioural changes needed for a healthy home.

## Data sharing

Access to clinical and entomological study data requires a formal application to the Scientific Coordinating Committee of the Medical Research Council Unit The Gambia (MRCG) and the Joint Gambian Government's and MRCG Ethics Committee in The Gambia at https://www.mrc.gm/scientific-coordinating-committee/

## Declaration of interests

We declare no competing interests.
