# Supplementary material for: Improved housing versus usual practice for additional protection against clinical malaria in The Gambia (RooPfs): a household-randomised controlled trial
Source: Lancet Planet Health. 2021 Apr 8;5(4):e220–9. doi: 10.1016/S2542-5196(21)00002-4 (PMC8051018; doi:10.1016/S2542-5196(21)00002-4)
Supplement: Supplementary appendix [file mmc1.pdf]

THE LANCET
Planetary Health

Supplementary appendix

This appendix formed part of the original submission and has been peer reviewed. We post it as supplied by the authors.

Supplement to: Pinder M, Bradley J, Jawara M, et al. Improved housing versus usual practice for additional protection against clinical malaria in The Gambia (RooPfs): a household-randomised controlled trial. *Lancet Planet Health* 2021; **5:** e220–29.

Supplementary material for Roo*Pf*s trial

Table S1: Vector control coverage

Data are % (n/N). Insecticide-treated net coverage is at least two nets/house.

Vector control	Unmodified houses	Modified houses
Insecticide-treated nets		
June 2016	54% (205/382)	51% (192/380)
July 2016	94% (360/382)	93% (355/380)
December 2016	98% (363/370)	98% (361/370)
June 2017	87% (313/360)	87% (319/365)
December 2017	96% (341/354)	91% (318/348)
Indoor residual spraying		
November 2016 (bendiocarb)	56% (126/227)	55% (119/218)
July 2017 (pirimiphos methyl)	86% (326/378)	89% (336/377)

Table S2: Full model results for primary endpoint: clinical malaria

CI=confidence intervals

Variable	Level	Effect estimate (95% CI)	p
Intervention	Unmodified houses	1	
	Modified houses	1.75 (1.15-2.65)	0.0085
Year	2016	1	
	2017	0.25 (0.15, 0.40)	<0.0001
River bank	North bank	1	
	South bank	0.53 (0.34-0.84)	0.0063
Month	July	1	
	August	1.62 (0.71-3.72)	0.25
	September	1.97 (0.85-4.59)	0.11
	October	4.67 (2.17-10.04)	<0.0001
	November	2.85 (1.30-6.24)	<0.0001
	December	1.14 (0.30-4.32)	0.84
Ethnicity	Non-Fula	1	
	Fula	1.46 (0.91-2.34)	0.12
Age	Age 0-4 years	1	
	Age 5-9 years	2.07 (1.24-3.47)	0.0054
	Age 10-14 years	2.16 (1.08-4.32)	0.029

Table S3: Full model results for density of *A. gambiae* s.l.
CI=confidence intervals

Variable	Level	Effect estimate (95% CI)	p
Intervention	Unmodified houses	1	
	Modified houses	1.23 (0.83, 1.81)	0.30
Year	2016	1	
	2017	0.22 (0.15, 0.31)	<0.0001
River bank	North bank	1	
	South bank	0.63 (0.44, 0.91)	0.015
Ethnicity	Non-Fula	1	
	Fula	1.23 (0.84, 1.79),	0.29
Incense (*churai*) burning	No	1	
	Yes	1.66 (0.96, 2.86)	0.068

Table S4: Entomological results Data are % (n/N) *Sporozoite analysis is odds ratio and EIR is a difference.

	Unadjusted estimates		Unadjusted rate ratio or odds ratio* (95% CI)	Adjusted rate ratio (95% CI)	p value
	Unmodified houses	Modified houses			
Light trap collections	708	732			
Mosquitoes collected	10,219	10,612			
Female *A. gambiae sensu lato* collected	2,330	2,635			
Species composition of *A. gambiae* sensu lato, 2016					
A. arabiensis	37% (251/674)	39% (277/514)			
A. gambiae sensu stricto	31% (211/674)	31% (222/514)			
A. coluzzii	32% (212/674)	30% (215/514)			
Species composition of *A. gambiae* sensu lato, 2017					
A. arabiensis	79% (274/348)	73% (447/613)			
A. gambiae sensu stricto	18% (63/348)	22% (137/613)			
A. coluzzii	3% (11/348)	5% (29/613)			
Female *A. gambiae* sensu lato per trap per night					
Overall	3 (2330/708)	4 (2635/732)	1.28 (0.87-1.89)	1.23 (0.83-1.81)	p=0.30
2016	6 (1982/354)	6 (2020/366)	0.99 (0.65-1.49)	0.98 (0.65-1.47)	p=0.92
2017	1 (348/354)	2 (615/366)	1.71 (0.93-3.14)	1.63 (0.96-2.75)	p=0.07
Percentage of mosquitoes with sporozoites					
Overall	0.3% (7/2317)	0.2% (5/2644)	0.63 (0.20–1.97)		p=0.42
2016	0.2% (3/1954)	0.1 % (1/2052)	0.32 (0.03-3.05)		p=0.32
2017	1% (4/363)	1% (4/582)	0.62 (0.15-2.50)		p=0.50
Estimated EIR (infective bites per transmission season)					
Overall	1.79	1.23	0.57 (-1.86-3.00)		p=0.65
2016	1.55	0.49	1.07 (-2.19-4.33)		p=0.52
2017	1.94	1.99	-0.05 (-3.10-3.00)		p=0.98
Other species of mosquitoes per trap per night					
Overall	10 (6854/708)	9 (6983/732)	0.99 (0.68-1.43)	0.99 (0.71-1.37)	p=0.94
2016	10 (3520/354)	10 (3576/366)	0.98 (0.62-1.56)	0.99 (0.69-1.43)	p=0.97
2017	9 (3334/354)	9 (3407/366)	0.99 (0.69-1.43)	0.99 (0.70-1.40)	p=0.95

Table S5: Integrity of study houses at the end of the study.

Data are % (n/N).

House characteristic	Unmodified houses	Modified houses
Eaves		
Eaves closed	2% (8/337)	100% (363/363)
Windows in gables		
Screening intact	No gables	96% (343/357)
Windows in house walls		
One window	16% (54/334)	28% (102/361)
Two windows	4% (12/334)	4% (13/361)
Screening intact	0% (0/66)	83% (95/115)
Front Door		
Gap between door and frame	91% (311/341)	24% (87/362)
Gap on all 4-sides of door	65% (223/345)	3% (10/363)
Netting intact	No netting	25% (90/363)
Back door		
Gap between door and frame	88% (304/345)	19% (67/363)
Gap on all 4-sides of door	69% (239/345)	1% (4/363)
Netting intact	No netting	76% (275/360)

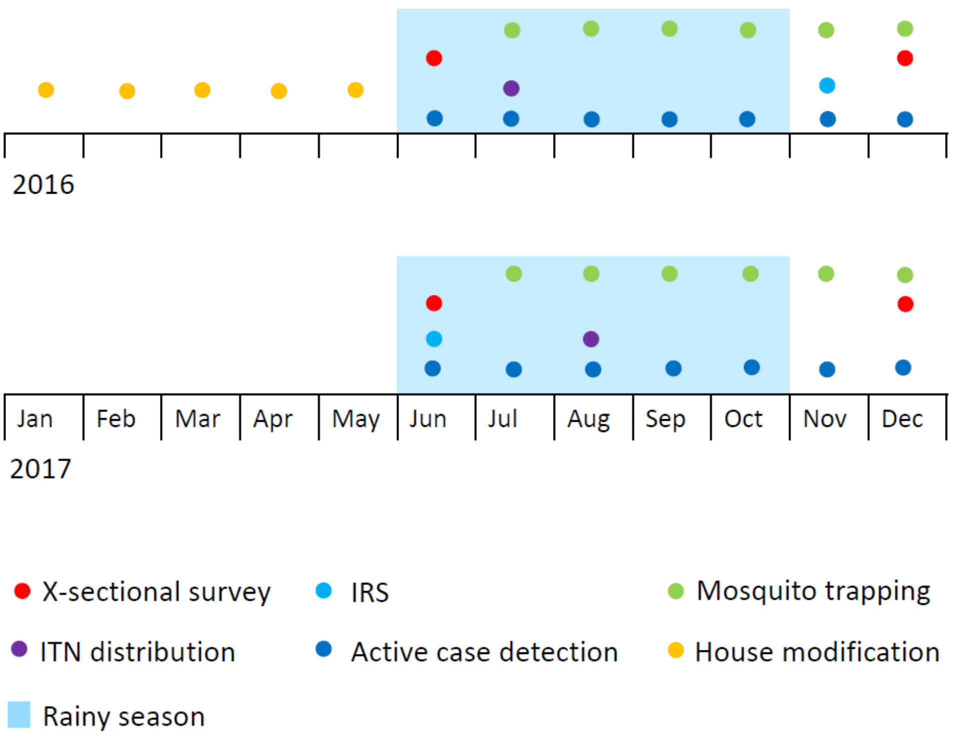

Figure S1: **Timings of interventions and outcome assessment**

X-sectional survey=cross-sectional survey, IRS=indoor residual spraying, ITN=insecticide-treated net.

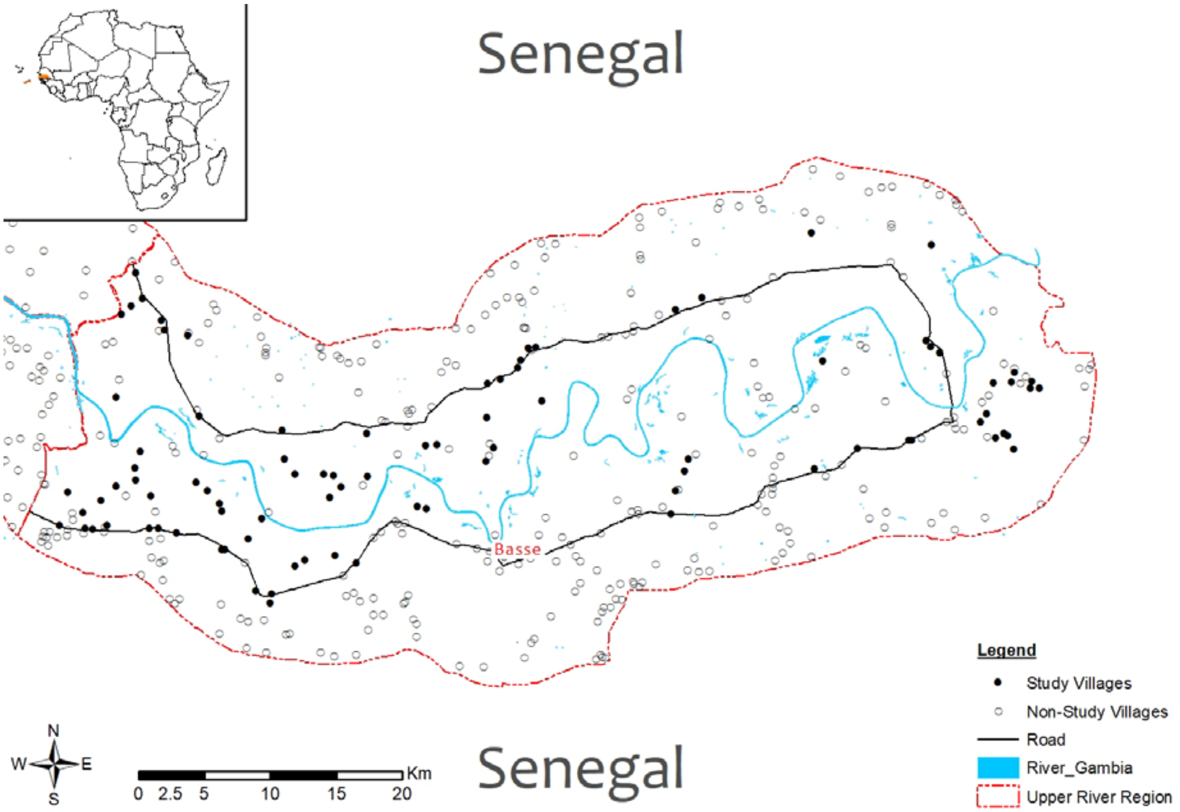

***Figure S2*: Study map**

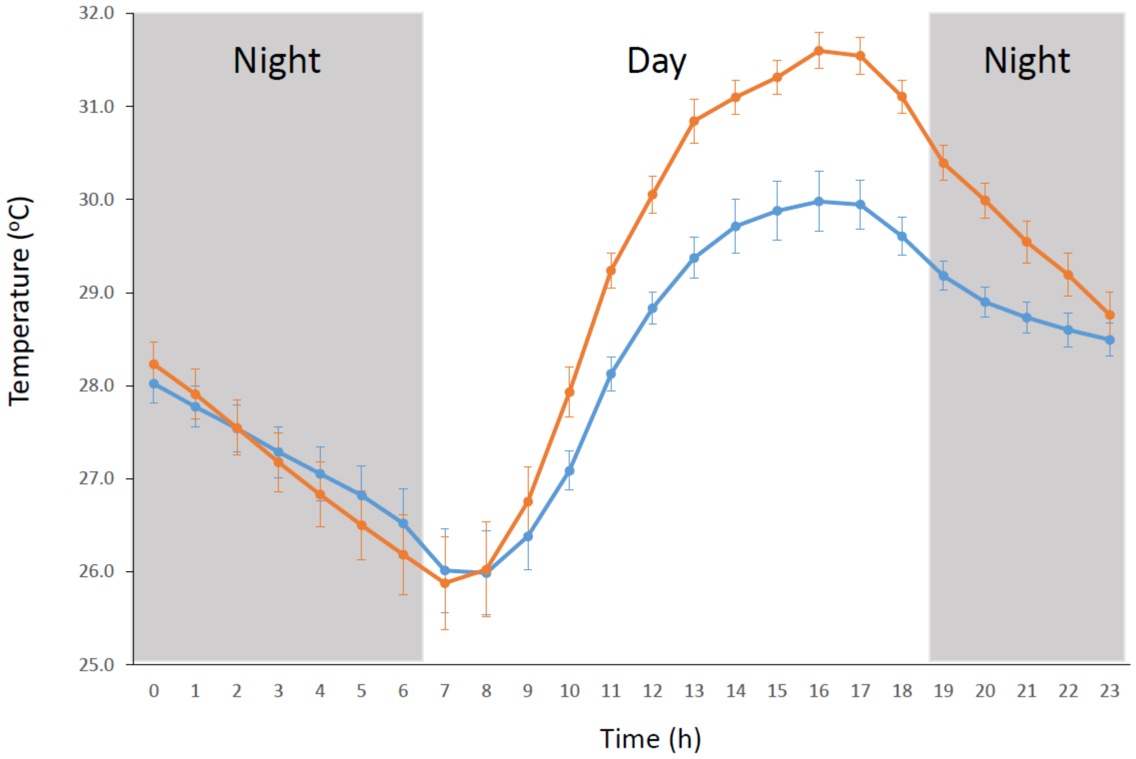

Figure 3: **Indoor temperature in study houses**

Changes in indoor temperature in standard thatched-roofed houses (n=15, blue) and metal-roofed screened houses (n=13, orange). Error bars are standard errors.
